# The Effects of House Cooking Process on Residue Concentrations of 41 Multi-Class Pesticides in Rice

**Published:** 2018

**Authors:** Attaollah Shakoori, Hassan Yazdanpanah, Farzad Kobarfard, Mohammad Hossein Shojaee, Jamshid Salamzadeh

**Affiliations:** a *Food Safety Research Center, Shahid Beheshti University of Medical Sciences, Tehran, Iran. *; b *Deputy for Food and Drug Affairs, Shahid Beheshti University of Medical Sciences, Tehran, Iran.*; c *Toxicology and Pharmacology Deptartment, School of Pharmacy, Shahid Beheshti University of Medical Sciences, Tehran, Iran. *; d *Phytochemistry Research Center, Shahid Beheshti University of Medical Sciences, Tehran, Iran. *; e *Medicinal Chemistry Deptartment, School of Pharmacy, Shahid Beheshti University of Medical Sciences, Tehran, Iran. *; f *Faroogh Life Sciences Research Laboratory, Tehran, Iran. *; g *Clinical Pharmacy Deptartment, School of Pharmacy, Shahid Beheshti University of Medical Sciences, Tehran, Iran.*

**Keywords:** Cooking process, Pesticide residues, Multi-residue analysis, LC–MS/MS, Rice

## Abstract

In current study, the effects of Iranian rice cooking method (Kateh) on residue levels of 41 pesticides were investigated. The pesticides were selected according to Iranian National Standards Organization (INSO) regulations and covered 18 permitted and 23 banned pesticides belonging to different chemical classes such as organophosphate, triazole, and carbamate. A 250 g portion of rice sample was soaked in 2.5 L spiked tap water containing studied pesticides at final concentration 2 μg/mL and then, the effects of washing and cooking were investigated. The pesticides were analyzed simultaneously in a single run using positive electrospray ionisation with multiple reaction monitoring (MRM) after extraction with QuEChERS method. The results showed that washing removed different portions of pesticide residues in the range between 12.0-88.1%. Washing effect was not associated with the water solubility of the pesticides but amount of residue binding to rice matrix was a major factor for residue reduction. In Iranian method of rice preparation, cooking process includes boiling and steam cooking. In this study, the amount of the pesticide residues was decreased in the range of 20.7-100%. Under these conditions, volatilization, hydrolysis, and thermal degradation caused the reduction of the pesticide residues.

## Introduction

Rice as a cereal crop, is the most important and principal staple food for a large group of the human population in the world. Rice is the major source of energy in 17 countries in Asia and the Pacific, nine countries in North and South America and eight countries in Africa. More than 3.5 billion people depend on rice for survival and it supplies 20% of the calories consumed worldwide. Although rice alone cannot supply all of the nutrients necessary for adequate nutrition, but it is a good source of thiamine, riboflavin, niacin, glutamic and aspartic acid. Unmilled rice contains a significant amount of dietary fiber (1). Botanically, rice is an annual plant and belongs to the genus Oryza that includes about 22 species. Only two species of rice are considered important as food species for humans and cultivated in the world: Oryza sativa is grown worldwide and Oryza glaberrima grown in parts of West Africa (2). In the recent decades, rice production has increased and according to FAO data, global production of rice has risen steadily from about 215 million tons of paddy rice in 1961 to over 738 million tons in 2012 (3). 

The use of pesticides, like pre and post-emergence herbicides, insecticides, and fungicides during various stages of cultivation is one of the most important factors associated with growing in rice production. However, the use of these pesticides affects the whole system of rice: the soil, water, and rice grain. In addition to commonly used pesticides, presence of banned pesticides in rice is another important challenge (4). Nowadays, appearance of pesticide residues in human foods including rice is a great challenge in food safety. Pesticides cause public concern due to their potential adverse effects on human health, which is most obvious in the developing fetus and young child (5). The most common route of exposure to pesticides is ingestion of treated food commodities containing residues. To ensure the safety of food for consumers, numerous legislations such as the EU directives (6) or the Iranian regulation (7), have established maximum residue limits (MRL) for pesticides in foodstuffs. Therefore, control and management of pesticide residues in foods, according to regulations require powerful analytical methods. For these reasons there is a clear need to develop fast methods for the multi-residue analysis of the most commonly used and banned pesticides in rice crops.

Although various techniques based on GC and LC methods coupled to the different detectors such as electron capture detector (ECD), nitrogen-phosphorus detector (NPD) and mass spectrometry detector (MS) have been developed for determination of pesticides residues in rice (8, 9), most of the are suitable for analysis of raw rice samples. Rice is cooked in different methods by boiling, steaming, or in a microwave oven around the world (10). Therefore, food safety investigators are interested to study the effects of cooking processes in pesticide residue levels in rice. 

Previous studies in different commodities showed that cooking processes reduced pesticide levels in home and industrial food preparation. In 2002, Lalah and Wandiga studied the effect of boiling on the removal of persistent malathion residues from cooked beans and maize. The results showed that malathion and its polar metabolites, malathion-α and malathionβ-monocarboxylic acids were completely removed by boiling, but malaoxon was not eliminated and still detected in high levels in the solvent extracts of cooked beans and maize (11). Effects of cooking in a microwave oven on elimination of triﬂuralin, chlorpyrifos, decamethrin, cypermethrin and dichlorvos in rice and beans were studied. The spiked rice and beans samples, at levels of 1.0 mg/kg, were cooked at powers of 500 W and 800 W for 15- 45 min, respectively. After cooking, the levels of spiked pesticides were decreased from 92% to 99% in both rice and beans samples (12). Shoeibi *et al.* investigated the effect of cooking on removal of three carbamates including carbaryl, propoxur and pirimicarb on spiked rice samples. The levels of pesticides reduced 78%, 55%, and 35% for carbaryl, propoxur, and pirimicarb, respectively, after cooking (13). Another study showed that combination of heat and water in house and industrial processing, dramatically reduced the levels of four fungicides includings, boscalid, mancozeb, iprodione, and propamocarb and insecticide deltamethrin in spinach (14). Angel Yang *et al. *monitored 44 multi-class pesticides in 31 different food materials using QuEChERS extraction and LC-MS/MS method. In nine samples including colored rice, glutinous rice (white rice), glutinous rice (unpolished rice), green chili, ginger, butterbur, chinamul, spinach, and perilla leaf, 8 pesticides including acetamiprid, azoxystrobin, fenobucarb, fosthiazate, iprobenfos, lufenuron, propiconazole, and triﬂoxystrobin were detected. After cooking and washing the positive samples, residue levels of mentioned compounds were considerably decreased (15).

The above mentioned studies confirmed that various cooking methods and in house preparation of foods can decrease the residue levels of some pesticides. Therefore, it is necessary to determine the reduction of other pesticide residues in different commodities that are processed in various methods all over the world. In the present study, we investigated the effects of the Iranian traditional in house method for rice cooking (Kateh) on reduction of 41 multi-class pesticide residues using QuEChERS extraction-based LC-ESI-MS/MS technique

## Experimental

*Chemicals*

Pesticides reference standards (purity > 96.0%), triphenylphosphate (TTP) as internal standard, and anhydrous magnesium sulfate (MgSO_4_) were purchased from Sigma–Aldrich/Fluka/Riedel-de-Haën (Germany). Ammonium formate, methanol (MeOH) and HPLC-grade acetonitrile (MeCN) were purchased from Acros (Belgium). Ethyl acetate (EtAc), glacial acetic acid (HOAc), and sodium acetate were supplied from Merck (Darmstadt, Germany). Bondesil-primary secondary amine (PSA, 40 μm) was provided from Interchim (France). HPLC grade water was obtained by purifying demineralized water on a Milli-Q Plus ultra-pure water system (Millipore, Molsheim, France).

Individual stock solutions of pesticides at a concentration of 1000 μg/mL were prepared in ethyl acetate (and methanol for Cartap and Fuberidazole) according to their solubility at 20 °C. A mixed intermediate standard solution at a concentration of 5 μg/mL was prepared via appropriate dilution of the stock solutions in MeOH containing 0.1% HOAc in order to avoid the degradation of the pesticides (8). This solution was used as a spiking solution for validation experiments. Matrix-matched multi-level calibration standards solutions were prepared using sample extracts obtained from organic rice. Aliquots of blank samples (5 mL of final MeCN layer), which were extracted via QuEChERS method were evaporated and reconstituted in 5 mL of mixture of appropriate working standard solutions and 0.02% HOAc in MeOH to generate ﬁnal concentrations of 0.02, 0.04, 0.10, 0.20, 0.50, and 1.0 mg/kg for the matrix-matched calibration standards. A stock solution of triphenylphosphate (TTP) in ethyl acetate at concentration of 20 μg/mL was used as internal standard and an aliquot of 50 μL of TTP solution in ethyl acetate (20 μg/mL) was added to the spiked rice sample. 

*Pesticide selection*

The 18 selected LC-amenable pesticides (carbaryl, cartap, chlorpyrifos, cinosulfuron, diazinon, edifenphos, malathion, oxadiazon, oxydemeton-methyl, primiphos-methyl, propiconazole, Spinosyn A and D, thiobencarb, thiophanate-methyl, triadimenol, tricyclazole, triflumizole) are used for rice production in Iran and MRLs have been established for them by Iranian National Standard Organization (INSO), NO.13120 (7). According to the regulation, some pesticides are banned to be used in Iran. Existence of banned pesticides in any kind of food including rice can produce health problems and it is necessary to investigate the presence of them in foods. Therefore, 23 banned LC-amenable pesticides according to INSO^ʼ^s list (5), including azinphos-ethyl, bromacil, carbofuran, chlorbromuron, chlorfenvinphos, coumaphos, dialifos, dicrotophos, etrimfos, fluometuron, fuberidazole, iprobenfos, methabenzthiazuron, methidathion, monocrotophos, omethoate, phosphamidon, phoxim, propoxur, pyrazophos, TCMTB, tri-allate, and triazophos were selected. 

The comprehensive list covers 41 pesticides with different modes of action such as herbicides, fungicides, insecticides and plant growth regulators with different chemical natures such as organophosphates, carbamates, Spinosyn A and D, strobilurins, and quaternary ammoniums (Table 1).

*Sample preparation for processing*

Before cooking process, 5 mL of mixed pesticides (1000 μg/mL) standard stock solution were dissolved in 2.5 L tap water (final concentration 2 μg/mL). Two-hundred and fifty grams of rice sample was submerged in the mentioned solutions containing the 41 pesticides followed by air-drying at room temperature in a hood for 24 h. A 50 g portion of the samples was ground with 50 g dry ice and analyzed as control sample and the rest was used for evaluation of different processing factors. For each process (including washing and cooking) 50 g rice was used. 

**Table 1:** Names, molar masses, MRM parameters, ion ratios and retention times of the studied pesticides for LC-MS/MS analysis

**No.**	**Pesticides**	**Molar mass**	**Precursor ion**	**CV (V)**	**1st Transition (quantitation)**	**CE (eV)**	**2nd Transition (conﬁrmation)**	**CE (eV)**	**Rt (min)**	**Ion ratio**
1	Azinphos-ethyl	345	(M+H)^+^	15	346⟶77	36	346⟶132	30	18.99	1.43
2	Bromacil	261	(M+H)^+^	20	261⟶205	12	261⟶188	35	13.75	7.03
3	Carbaryl	201	(M+H)^+^	15	202⟶145	20	202⟶117	10	14.61	3.85
4	Carbofuran	221	(M+H)^+^	15	222⟶165	16	222⟶123	16	15	1.4
5	Cartap	237	(M+H)^+^	27	238⟶73	16	238⟶150	16	2.57	1.75
6	Chlorbromuron	292	(M+H)^+^	28	293⟶204	16	293⟶182	16	18.47	1.3
7	Chlorfenvinphos	358	(M+H)^+^	28	359⟶99	28	359⟶155	17	21.05	2.11
8	Chlorpyrifos	350	(M+H)^+^	30	350⟶97	25	350⟶198	22	25.24	2.08
9	Cinosulfuron	413	(M+H)^+^	16	414⟶183	15	414⟶157	15	7.06	17.9
10	Coumaphos	362	(M+H)^+^	35	363⟶307	17	363⟶289	25	20.95	3.23
11	Dialifos	393	(M+H)^+^	20	394⟶187	10	394⟶208	15	22.77	1.12
12	Diazinon	304	(M+H)^+^	29	305⟶97	35	305⟶169	20	21.92	1.88
13	Dicrotophos	237	(M+H)^+^	26	238⟶112	10	238⟶193	10	4.32	3.34
14	Edifenphos	310	(M+H)^+^	30	311⟶109	32	311⟶111	26	20.5	4.85
15	Etrimfos	292	(M+H)^+^	35	293⟶125	25	293⟶265	18	21.67	2.66
16	Fluometuron	232	(M+H)^+^	30	233⟶72	18	233⟶46	20	15.33	4.87
17	Fuberidazole	184	(M+H)^+^	42	185⟶157	25	185⟶156	32	12.21	2.24
18	Iprobenfos	288	(M+H)^+^	20	289⟶91	18	289⟶205	14	20.19	6.68
19	Malathion	330	(M+H)^+^	18	331⟶127	12	331⟶99	20	18.43	1.44
20	Methabenzthiazuron	221	(M+H)^+^	28	222⟶165	20	222⟶150	30	15.53	2.16
21	Methidathion	302	(M+H)^+^	18	303⟶145	20	303⟶85	10	16.93	1.23
22	Monocrotophos	223	(M+H)^+^	26	224⟶127	18	224⟶98	14	3.73	2.25
23	Omethoate	213	(M+H)^+^	20	214⟶125	18	214⟶183	15	3.1	4.35
24	Oxadiazon	344	(M+H)^+^	30	345⟶220	13	345⟶177	40	24.27	1.49
25	Oxydemeton-methyl	246	(M+H)^+^	20	247 ⟶109	25	247 ⟶169	14	3.26	1.28
26	Phosphamidon	299	(M+H)^+^	26	300⟶127	20	300⟶174	10	12.47	2.71
27	Phoxim	298	(M+H)^+^	16	299⟶129	13	299⟶153	11	21.46	5.91
28	Primiphos-methyl	305	(M+H)^+^	30	306⟶108	28	306⟶164	17	22.44	4.76
29	Propiconazole	341	(M+H)^+^	40	342⟶159	30	342⟶69	16	21.26	1.04
30	Propoxur	209	(M+H)^+^	20	210⟶111	14	210⟶168	8	13.93	3.23
31	Pyrazophos	373	(M+H)^+^	36	374⟶222	30	374⟶194	21	22.13	1.3
32	Spinosyn A	732	(M+H)^+^	53	733⟶142	30	733⟶98	56	26.65	3.72
33	Spinosyn D	746	(M+H)^+^	50	747⟶142	31	747⟶98	51	27.44	4.36
34	TCMTB	238	(M+H)^+^	21	239⟶180	10	239⟶136	40	18.1	3.6
35	Thiobencarb	257	(M+H)^+^	18	258⟶125	20	258⟶100	13	22.57	7.66
36	Thiophanate-methyl	342	(M+H)^+^	24	343⟶151	22	343⟶311	12	13.51	6.06
37	Triadimenol	295	(M+H)^+^	20	296⟶70	8	296⟶99	15	19.66	7.43
38	Tri-allate	303	(M+H)^+^	32	304⟶86	20	304⟶143	24	25.34	1.08
39	Triazophos	313	(M+H)^+^	31	314⟶162	18	314⟶119	32	18.85	1.8
40	Tricyclazole	189	(M+H)^+^	38	190⟶136	27	190⟶163	22	10.4	1.41
41	Triflumizole	345	(M+H)^+^	10	346⟶278	8	346⟶73	25	23.17	2.36
42	Triphenylphosphate*	326	(M+H)^+^	20	327⟶77	45	327⟶152	45	20.81	1.94

* Internal standard.

**Table 2:** Mean recoveries (%) and relative standard deviations, RSDr (%), LOQs and LODs (mg/kg) obtained for 41 compounds in rice samples, spiked at 0.025, 0.250 and 1.000 mg/kg levels (n = 5)

**NO.**	**Compound**	**0.025 mg/kg**		**0.250 mg/kg**		**1.000 mg/kg**		**LOQ** **(mg/kg)**	**LOD** **(mg/kg)**
		**Mean**	**RSDr**	**Mean**	**RSDr**	**Mean**	**RSDr**		
1	Azinphos-ethyl	90	4	85	8	81	2	0.015	0.005
2	Bromacil	104	5	79	5	81	8	0.016	0.005
3	Carbaryl	96	5	84	8	81	3	0.009	0.003
4	Carbofuran	81	8	82	7	84	9	0.012	0.004
5	Cartap	92	18	101	16	99	9	0.015	0.005
6	Chlorbromuron	88	15	84	7	95	6	0.016	0.005
7	Chlorfenvinphos	83	2	96	11	94	9	0.018	0.006
8	Chlorpyrifos	111	2	81	7	82	10	0.019	0.006
9	Cinosulfuron	86	7	91	18	91	7	0.013	0.004
10	Coumaphos	98	10	105	11	87	5	0.019	0.006
11	Dialifos	116	3	103	9	87	9	0.014	0.005
12	Diazinon	109	1	98	4	101	11	0.016	0.005
13	Dicrotophos	94	13	92	11	98	8	0.013	0.004
14	Edifenphos	72	3	95	10	89	8	0.014	0.005
15	Etrimfos	103	3	92	8	96	7	0.016	0.005
16	Fluometuron	77	3	94	10	97	6	0.012	0.004
17	Fuberidazole	109	6	85	12	79	9	0.017	0.006
18	Iprobenfos	92	5	95	10	96	10	0.012	0.004
19	Malathion	90	4	90	16	94	9	0.013	0.004
20	Methabenzthiazuron	99	8	81	6	77	6	0.014	0.005
21	Methidathion	110	5	98	10	93	7	0.019	0.006
22	Monocrotophos	79	16	101	5	103	6	0.014	0.005
23	Omethoate	100	14	93	12	98	10	0.014	0.005
24	Oxadiazon	100	3	100	6	94	10	0.014	0.005
25	Oxydemeton-methyl	88	18	110	7	115	5	0.014	0.005
26	Phosphamidon	103	6	77	8	87	10	0.015	0.005
27	Phoxim	78	12	102	14	100	6	0.014	0.005
28	Primiphos-methyl	117	3	96	3	101	8	0.020	0.007
29	Propiconazole	107	3	87	6	89	12	0.012	0.004
30	Propoxur	75	7	86	18	83	12	0.017	0.006
31	Pyrazophos	108	2	92	9	90	3	0.010	0.003
32	Spinosyn A	102	9	94	17	100	18	0.014	0.005
33	Spinosyn D	84	13	92	12	95	7	0.016	0.005
34	TCMTB	104	4	81	13	84	13	0.014	0.005
35	Thiobencarb	104	4	100	10	95	8	0.015	0.005
36	Thiophanate-methyl	81	6	110	12	92	14	0.016	0.005
37	Triadimenol	78	1	76	8	94	7	0.012	0.004
38	Triallate	75	5	80	14	78	5	0.013	0.004
39	Triazophos	98	9	81	15	81	9	0.011	0.004
40	Tricyclazole	89	4	82	15	79	10	0.016	0.005
41	Triflumizole	79	12	84	13	95	12	0.010	0.003

**Table 3:** Mean concentrations (± SD, n = 3), mean values of processing factors (PF) and reductions (%) of the pesticides in unprocessed rice samples, after washing and cooking

No.	Compounds	** Unprocessed samples**	Washing			Cooking		
		Concentration (mg/kg) (mean ± SD)	Concentration (mg/kg) (mean ± SD)	PF	Reduction (% )	Concentration (mg/kg) (mean ± SD)	PF	Reduction (% )
**1**	Azinphos-ethyl	0.880 ( ± 0.071 )	0.755 ( ± 0.044 )^*^	0.86	14.2	0.096 ( ± 0.034 )	0.11	89.1
**2**	Bromacil	0.961 ( ± 0.029 )	0.649 ( ± 0.094 )	0.68	32.5	0.179 ( ± 0.016 )	0.19	81.4
**3**	Carbaryl	0.880 ( ± 0.031 )	0.668 ( ± 0.069 )	0.76	24.1	0.195 ( ± 0.018)	0.22	77.9
**4**	Carbofuran	0.905 ( ± 0.086 )	0.878 ( ± 0.058 )^*^	0.97	3.0	0.505 ( ± 0.081 )	0.56	44.2
**5**	Cartap	0.842 ( ± 0.034 )	0.540 ( ± 0.016 )	0.64	35.8	0.020 ( ± 0.064 )	0.02	97.6
**6**	Chlorbromuron	0.982 ( ± 0.017 )	0.603 ( ± 0.076 )	0.61	38.6	0.498 ( ± 0.083 )	0.51	49.2
**7**	Chlorfenvinphos	0.983 ( ± 0.047 )	0.637 ( ± 0.088 )	0.65	35.2	0.286 ( ± 0.048 )	0.29	71.0
**8**	Chlorpyrifos	0.855 ( ± 0.078 )	0.409 ( ± 0.038 )	0.48	52.2	0.163 ( ± 0.018 )	0.19	80.9
**9**	Cinosulfuron	0.904 ( ± 0.088 )	0.871 ( ± 0.061 )^*^	0.96	3.6	0.669 ( ± 0.031 )	0.74	26.0
**10**	Coumaphos	0.897 ( ± 0.030 )	0.837 ( ± 0.108 )^*^	0.93	6.7	0.711 ( ± 0.074 )	0.79	20.7
**11**	Dialifos	0.909 ( ± 0.049 )	0.551 ( ± 0.071 )	0.61	39.4	0.385 ( ± 0.039 )	0.42	57.6
**12**	Diazinon	0.982 ( ± 0.0.25 )	0.492 ( ± 0.060 )	0.50	49.9	0.557 ( ± 0.069 )	0.57	43.3
**13**	Dicrotophos	0.956 ( ± 0.062 )	0.242 ( ± 0.083 )	0.25	74.7	0.349 ( ± 0.038 )	0.37	63.5
**14**	Edifenphos	0.921 ( ± 0.078 )	0.648 ( ± 0.073 )	0.70	29.7	0.730 ( ± 0.045 )	0.79	20.7
**15**	Etrimfos	0.996 ( ± 0.017 )	0.957 ( ± 0.023 )^*^	0.96	3.9	0.499 ( ± 0.093 )	0.50	49.9
**16**	Fluometuron	0.903 ( ± 0.109 )	0.837 ( ± 0.050 )*	0.93	7.3	0.205 ( ± 0.084 )	0.23	77.3
**17**	Fuberidazole	0.890 ( ± 0.107 )	0.194 ( ± 0.075 )	0.22	78.2	0.119 ( ± 0.067 )	0.13	86.7
**18**	Iprobenfos	0.806 ( ± 0.042 )	0.476 ( ± 0.047 )	0.59	41.0	0.408 ( ± 0.091 )	0.51	49.3
**19**	Malathion	0.982 ( ± 0.031 )	0.864 ( ± 0.010 )	0.88	12.0	0.260 ( ± 0.004 )	0.26	73.6
**20**	Methabenzthiazuron	0.926 ( ± 0.082 )	0.468 ( ± 0.086 )	0.51	49.5	0.331 ( ± 0.087 )	0.36	64.3
**21**	Methidathion	0.958 ( ± 0.065 )	0.641 ( ± 0.067 )	0.67	33.1	0.242 ( ± 0.047 )	0.25	74.7
**22**	Monocrotophos	0.767 ( ± 0.024 )	0.562 ( ± 0.091 )	0.73	26.7	0.350 ( ± 0.027 )	0.46	54.4
**23**	Omethoate	0.783 ( ± 0.098 )	0.284 ( ± 0.048 )	0.36	63.7	0.339 ( ± 0.029 )	0.43	56.7
**24**	Oxadiazon	0.979 ( ± 0.089 )	0.117 ( ± 0.115 )	0.12	88.1	0.216 ( ± 0.060 )	0.22	77.9
**25**	Oxydemeton-methyl	0.793 ( ± 0.035 )	0.183 ( ± 0.061 )	0.23	76.9	<LOQ	0.00	99.0
**26**	Phosphamidon	0.911 ( ± 0.035 )	0.677 ( ± 0.082 )	0.74	25.7	0.474 ( ± 0.053 )	0.52	48.0
**27**	Phoxim	1.012 ( ± 0.074 )	0.615 ( ± 0.030 )	0.61	39.2	0.722 ( ± 0.034 )	0.71	28.6
**28**	Primiphos-methyl	1.019 ( ± 0.109 )	0.793 ( ± 0.097 )*	0.78	22.1	0.139 ( ± 0.056 )	0.14	86.4
**29**	Propiconazole	0.935 ( ± 0.092 )	0.064 ( ± 0.041 )	0.07	87.1	0.432 ( ± 0.096 )	0.46	53.8
**30**	Propoxur	0.885 ( ± 0.021 )	0.586 ( ± 0.092 )	0.66	33.7	0.202 ( ± 0.009 )	0.23	77.2
**31**	Pyrazophos	0.910 ( ± 0.014 )	0.416 ( ± 0.007 )	0.46	54.3	0.502 ( ± 0.036 )	0.55	44.8
**32**	Spinosyn A	1.110 ( ± 0.057 )	0.586 ( ± 0.043 )	0.53	47.2	0.344 ( ± 0.070 )	0.31	69.0
**33**	Spinosyn D	0.975 ( ± 0.064 )	0.413 ( ± 0.103 )	0.42	57.6	0.226 ( ± 0.087 )	0.23	76.9
**34**	TCMTB	0.905 ( ± 0.007 )	0.213 ( ± 0.021 )	0.24	76.5	0.067 ( ± 0.041 )	0.07	92.6
**35**	Thiobencarb	0.990 ( ± 0.042 )	0.300 ( ± 0.002 )	0.30	69.7	0.505 ( ± 0.036 )	0.51	49.0
**36**	Thiophanate-methyl	1.000 ( ± 0.028 )	0.241 ( ± 0.024 )	0.24	75.9	0.299 ( ± 0.057)	0.30	70.1
**37**	Triadimenol	0.912 ( ± 0.054 )	0.462 ( ± 0.036 )	0.51	49.3	0.258 ( ± 0.061 )	0.28	71.7
**38**	Triallate	0.800 ( ± 0.028 )	0.491 ( ± 0.096 )	0.61	38.6	0.279 ( ± 0.013 )	0.35	65.1
**39**	Triazophos	0.850 ( ± 0.014 )	0.223 ( ± 0.005 )	0.26	73.8	0.359 ( ± 0.090 )	0.42	57.8
**40**	Tricyclazole	0.835 ( ± 0.021 )	0.229 ( ± 0.044 )	0.27	60.6	0.409 ( ± 0.041 )	0.49	51.1
**41**	Triflumizole	1.001 ( ± 0.028 )	0.357 ( ± 0.040 )	0.36	64.3	0.484 ( ± 0.093 )	0.48	51.6

* Values are not significantly different (*p* > 0.05).

**Table 4:** Physico-chemical properties of the pesticides studied in this investigation (according to the references 2-6).

**No.**	**Compound**	**Molecular formula**	**Chemical group**	**Pesticide type**	**Melting Point (°C)**	**log P (octanol-water)**	**Water solubility (mg L-1) at 25 °C**	**Vapour Pressure (Pa) at 25 °C**
1	Azinphos-ethyl^b^	C12H16N3O3PS2	Organophosphate	Insecticide, Acaricide	53	3.4	10.5	1.80E-08
2	Bromacil^b^	C9H13BrN2O2	Uracil	Herbicide	158	2.11	815	2.30E-09
3	Carbaryl^a^	C12H11NO2	Carbamate	Insecticide, Plant growth regulator	145	2.36	110	1.02E-08
4	Carbofuran^b^	C12H15NO3	Carbamate	Insecticide, Nematicide, Acaricide, Metabolite	151	2.32	320	3.64E-08
5	Cartap^a^	C7H15N3O2S2	Unclassified	Insecticide	131	-0.95	2.0 E 05	7.50E-13
6	Chlorbromuron^b^	C9H10BrClN2O2	Urea	Herbicide	96	3.09	35	2.98E-09
7	Chlorfenvinphos^b^	C12H14Cl3O4P	Organophosphate	Insecticide, Acaricide, Veterinary treatment	-20	3.81	124	5.63E-08
8	Chlorpyrifos^a^	C9H11Cl3NO3PS	Organophosphate	Insecticide	42	4.96	1.12	1.52E-07
9	Cinosulfuron^a^	C15H19N5O7S	Sulfonylurea	Herbicide	130	2.04	120	5.19E-15
10	Coumaphos^b^	C14H16ClO5PS	Organothiophosphates	Insecticide, Miticide	93	4.13	1.5	7.28E-10
11	Dialifos^b^	C14H17ClNO4PS2	Organophosphate	Insecticide, Acaricide	68	4.69	0.18	4.65E-10
12	Diazinon^a^	C12H21N2O3PS	Organophosphate	Insecticide, Acaricide, Repellent, Veterinary treatment	< 25	3.81	40	6.76E-07
13	Dicrotophos^b^	C8H16NO5P	Organophosphate	Insecticide	< 25	-0.49	1.00E+06	1.20E-06
14	Edifenphos^a^	C14H15O2PS2	Organophosphate	Fungicide	< 25	3.48	56	2.03E-09
15	Etrimfos^b^	C10H17N2OPS	Organophosphate	Insecticide	-3.35	2.94	40	6.00E-07
16	Fluometuron^b^	C10H11F3N2O	Phenylurea	Herbicide	164	2.42	110	7.04E-09
17	Fuberidazole^b^	C11H8N2O	Benzimidazole	Fungicide	292	2.67	71	5.06E-11
18	Iprobenfos^b^	C13H21O3PS	Organophosphate	Fungicide	< 25	3.34	400	3.04E-07
19	Malathion^a^	C10H19O6PS2	Organophosphate	Insecticide, Acaricide, Veterinary treatment	2.8	2.36	143	2.54E-08
20	Methabenzthiazuron^b^	C10H11N3OS	Urea	Herbicide	120	2.64	59	8.48E-10
21	Methidathion^b^	C6H11N2O4PS3	Organophosphate	Insecticide, Acaricide	39	2.2	187	2.53E-08
22	Monocrotophos^b^	C7H14NO5P	Organophosphate	Insecticide, Acaricide	55	-0.2	8.18+05	1.64E-08
23	Omethoate^b^	C5H12NO4PS	Organophosphate	Insecticide, Acaricide, Metabolite	-28	-0.74	1.00E+06	1.86E-07
24	Oxadiazon^a^	C15H18Cl2N2O3	Oxidiazole	Herbicide	90	4.8	0.7	8.40E-10
25	Oxydemeton-methyl^a^	C6H15O4PS2	Organophosphate	Insecticide	-20	-0.74	1.00E+06	2.14E-07
26	Phosphamidon^b^	C10H19ClNO5P	Organophosphate	Insecticide, Acaricide	-45	0.79	1.00E+06	1.24E-07
27	Phoxim^b^	C12H15N2O3PS	Organophosphate	Insecticide, Disinfectant	6.1	4.39	4.1	1.19E-07
28	Pirimiphos-methyl^a^	C11H20N3O3PS	Organophosphate	Insecticide, Acaricide	15	4.2	8.6	1.13E-07
29	Propiconazole^a^	C15H17Cl2N3O2	Triazole	Fungicide	< 25	3.72	110	7.50E-09
30	Propoxurb	C11H15NO3	Carbamate	Insecticide, Acaricide, Veterinary treatment	87	1.52	1860	7.26E-08
31	Pyrazophos^b^	C14H20N3O5PS	Phosphorothiolate	Fungicide	51	3.8	4.2	7.35E-10
32	Spinosyn A^a^	C41H65NO10	Micro-organism derived	Insecticide, Veterinary treatment	84-99.5	4	235	3.00E-05
33	Spinosyn D^a^	C42H67NO10	Micro-organism derived	Insecticide, Veterinarytreatment	161.5-170	4.5	0.33	2.00E-05
34	TCMTB^b^	C9H6N2S3	Mercaptobenzothiazole	Fungicide, Microbiocide, Wood preservative	Liquid	3.3	125	2.34E-09
35	Thiobencarb^a^	C12H16ClNOS	Thiocarbamate	Herbicide	3.3	3.4	28	1.65E-07
36	Thiophanate-methyl^a^	C12H14N4O4S2	Benzimidazole	Fungicide	172	1.4	26.6	5.35E-10
37	Triadimenol^a^	C14H18ClN3O2	Triazole	Fungicide	124	2.9	120	2.33E-12
38	Triallate^c^	C10H16Cl3NOS	Thiocarbamate	Herbicide	29	4.6	4	9.00E-07
39	Triazophos^b^	C12H16N3O3PS	Organophosphate	Insecticide, Acaricide, Nematicide	3.5	3.34	39	2.18E-08
40	Tricyclazole^a^	C9H7N3S	Triazolobenzothiazole	Fungicide	187	1.7	1600	1.50E-09
41	Triflumizole^a^	C15H15ClF3N3O	Imidazole	Fungicide	63.5	1.4	1.25E+04	1.05E-08

a Permitted pesticides in Iran for rice production.

b Prohibited pesticides in Iran for rice production.

**Figure 1:**
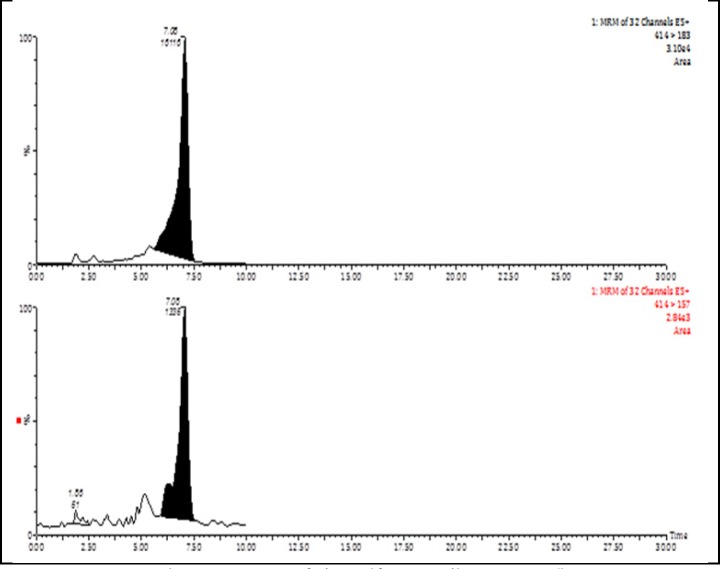
MRM Chromatograms of cinosulfuron (spike: 0.05 mg/kg

**Figure 2:**
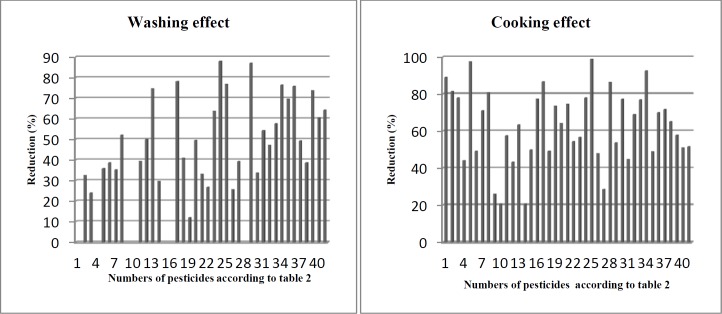
Effects of washing and cooking on pesticides residues in rice

*Cooking rice in Iranian traditional method (Kateh)*

*Washing*

A 50 g portion of the rice samples was completely washed twice with tap water and soaked in water. After 20 min, the samples were ground with dry ice and then analyzed. 

*Cooking*

For each sample, a mixture of 50 g of the rice sample, 50 mL of water, 2 g NaCl and 4 g edible oil were placed in a container. The mixture was boiled on a stove until the water was evaporated. Then, the lid of the container was completely closed and the flame of the stove minimized and rice sample steamed for 20 min. Then, the cooked rice sample were completely crushed and analyzed. 

*Extraction*

Extraction was performed by the original QuEChERS method (16). Five g of homogenized rice sample was accurately weighed into a 50 mL centrifuge tube. Appropriate concentrations of the mixed working standard solution (for spiking) and internal standard (TTP) were added to the tube and 10 mL of acetonitrile (MeCN) was added. The mixture was mixed using a vortex for 2.0 min, followed by addition of a mixture of 4 g anhydrous MgSO4 and 1.5 g sodium acetate and mixed using a vortex for 2.0 min again. The mixture was centrifuged for 5 min at 5433×g, and 5 mL of the supernatants then transferred into an appropriate tube placed in a nitrogen evaporator and evaporated at 40 °C until dryness. The residue was reconstituted in 0.5 mL MeCN. The mixture was mixed using a vortex for 2.0 min followed by sonication for 4.0 min and the solution was transferred to a tube containing 60 mg anhydrous MgSO_4_ and 20 mg primary secondary amine (PSA). The mixture was mixed using a vortex vigorously for 2 min and centrifuged for 5 min at 5433×g. Finally, a 0.4 mL aliquot of the cleaned extract was transferred into a screw cap vial and 100 μL of the solution injected into LC-MS/MS.

*Liquid chromatography*

The separation of the different pesticides from the samples was carried out using an Alliance separations module 2695 (Waters, Milford, MA, USA), which consist of a quaternary solvent delivery system, degasser, autosampler, column heater, and diode array detector coupled with a Quattro Micro Triple Quadrupole LC/MS (Waters, Micromass, Manchester, UK).

Chromatographic separation was performed using an Agilent ZORBAX Eclipse XDB-C_18_ (Narrow-Bore 2.1 × 150 mm, 3.5-micron) analytical column at a ﬂow rate of 0.2 mL/min and an injection volume of 100 μL. The mobile phase was 5 mM ammonium formate in methanol (solvent A) and 5 mM ammonium formate in water (solvent B) in a gradient mode and a total analysis time of 30 min. The elution program was as follows: at the start 30% solvent A and 70% solvent B; the percentage of solvent A was linearly increased to 100% in 20 min, then constant for 5 min and ramped to original composition in 5 min. The temperature of the column heater was maintained at 40 °C.

*Mass spectrometry*

The MS/MS system consisting of a triple quadrupole mass spectrometer Quattro Micro (Waters-Micromass, UK) was equipped with an electrospray source (Z-spray) and operated in positive ionization mode. MassLynx software, version 4.0, was used for instrument control and data acquisition. Analysis was performed in positive ion mode. The ESI source values were as follow: capillary voltage: 4.12 kV; extractor: 2 V; RF lens: 0.1 V; source temperature: 120 °C; desolvation temperature: 300 °C; and desolvation gas and cone gas (nitrogen 99.99% purity) ﬂow rates: 500 and 50 L/h, respectively. The analyzer settings were as follow: resolution: 14.6 (unit resolution) for LM1 and LM2 resolution and 14 for HM1 and HM2 resolution; ion energy 1 and 2, 0.3 and 3.0, respectively; entrance and exit energies: 55 and 75 (V); multiplier: 700 (V); and collision gas (argon, 99.995%) pressure: 5.35 × 10^-3 ^mbar.

MS/MS conditions for all pesticides were conducted in the positive electrospray ionization mode using multiple reaction monitoring (MRM) with two mass transitions. The optimization of the precursor ion, product ions, cone voltage and collision energy was performed via direct injection of the individual pesticide standard solution (1 μg/mL) into the mass spectrometer using a syringe pump at flow rate 10 μL/min. The product ion with the strongest intensity was used for quantitation, while the other with the lowest intensity was employed for conﬁrmation 

(Figure 1). The optimized parameters are presented in Table 1.

*Validation studies*

The validation study was performed based on the European SANCO guidelines (17). Linearity was studied using matrix-matched calibration curve by analyzing in triplicate six concentration levels, between 0.02 and 1.0 mg/kg. For determination of mean recoveries and precision five spiked blank rice samples at concentration levels of 0.025, 0.250, and 1.000 mg/kg were prepared and then treated according to the procedure described in sample preparation. The limit of quantiﬁcation (LOQ) was established as the lowest validated spike level meeting the method performance acceptability criteria (mean recoveries in the range 70-120%, with an RSDr ≤ 20%), and the limits of detection (LOD) estimated by a signal-noise ratio. The concentration of pesticides were determined by interpolation of the relative peak areas for each pesticide to internal standard peak area in the sample on the matrix-matched calibration curve. In order to compensate for losses during sample processing and instrumental analysis, internal standard (TPP) was used. Quality control samples were prepared using cooked rice at three concentration levels (0.025, 0.250, and 1.000 mg/kg) and validation parameters were calculated for them. All figures of merits were within the acceptable limits. 

*Statistical analysis*

Pesticide residue reduction during processing was evaluated by calculating the processing factor (PF) according to the equation PF = Mean C_after_/Mean C_before_, where C_after_ and C_before_ are the pesticide residue levels after and before processing (18). Final Data of residue levels were presented as mean ± standard deviation (SD), which were statistically evaluated by t-test analysis with Excel software. When residues were below limit of quantification (LOQ) after processing, the PF value was set as zero.

## Results and Discussion

*Method validation*

 Calibration curves were obtained by analyzing in triplicate six concentration levels, between 0.01 and 1.25 mg/kg. The range of coefficient of determinations (r^2^) was between 0.993 and 0.999. As shown in Table 2, mean recoveries ranged from 71-119% with satisfactory precision (RSDr < 20%). The limits of quantiﬁcation (LOQ) and the limits of detection (LOD) ranged between 0.010-0.025 mg/kg and 0.003-0.008 mg/kg, respectively.

*Unprocessed rice samples*

 For calculating the amount of pesticide reduction during the washing and cooking processes, it is necessary to determine the concentration of pesticides in unprocessed rice samples. All mean concentrations (three replicate) of the studied compounds in control raw samples and after washing and cooking are shown in Table 3. The mean concentration of the studied pesticides was in the range of 0.767-1.110 mg/kg. The obtained results are in accordance with OECD guideline (>0.1 m/kg), so that various processing factors can be determined (18). 

*Effects of washing*

 Washing is the most common form and generally the first step in household food processing. This study showed that washing removed different portions of pesticide residues. As shown in Table 3 and Figure 2, washing process removed the residues of 34 pesticides in the range between 12.0-88%, whereas azinphos-ethyl (organophosphate), carbofuran (carbamate), cinosulfuron (sulfonylurea), coumaphos (organothiophosphate), etrimfos (organophosphate), fluometuron (phenylurea), and primiphos-methyl (phosphorothioate) did not significantly get removed by washing. The most reduction occurred in oxadiazon (88.1%) and propiconazole (87.1%) that chemically belong to oxidiazole and triazole groups, respectively. On the other hand, the least removal occurred in the levels of malathion (12%), carbaryl (24.1%), and phosphamidon (25.7%). Our findings indicated that there was no correlation between chemical structure and the levels of residue removed by washing. For example in organophosphate group, the residues of etrimfos was not significantly decreased, while phoxim, omethoate, and oxydemeton-methyl were decreased 39.2% and 63.7%, and 76.9%, respectively. Therefore, we suggest that the loosely attached pesticides at surface of rice matrix were easily removed by washing. Our findings are consistent with previous studies. Angel Yang *et al.* (19), Chavarri *et al.* (20), and Kaushik *et al.* (21) suggested that the loosely attached residues were simply washed and removed from the surface of polluted food samples. We also found that there is no correlation between water solubility and residue removed by washing. For example, the residues of oxadiazon and spinosyn D with water solubility 0.70 and 0.33 g/mL were decreased 88.1% and 57.6%, respectively, while the reduction of phosphamidon and monocrotophos with water solubility 1.00E+06 and 8.18+05 g/mL was 25.7% and 26.7%, respectively (Tables 3 and 4). Previously, Cabras *et al.* (22, 23) and Walter *et al.* (24) indicated that water solubility is not a principal agent for removing pesticide residues by washing. Kaddus Miah *et al.* demonstrated that rice has high capacity for absorbing water during soaking (25). In this study, rice samples were soaked in contaminated water with different pesticides. Therefore, we propose that some very soluble pesticides such as phosphamidon and monocrotophos deeply penetrate to rice seeds and washing process cannot completely remove the residues.

*Effects of cooking*

 As shown in Table 3, the amounts of the pesticide residues decreased in range 20.7-100%. Oxydemeton-methyl (organophosphate) completely was removed (<LOQ) and the lowest removal occurred for coumaphos (organothiophosphate) and edifenphos (organophosphate). The residue reductions were not associated with the chemical group which the pesticide belong to. For example, in organophosphate group the concentration of edifenphos, diazinon, dicrotophos, and oxydemeton-methyl was decreased 20.7%, 43.3% 63.5%, and 100%, respectively. This controversial reduction was observed in other groups such as carbamates. Cooking rice in Iranian method (Kateh) consists of two principal steps; boiling and steam cooking. During boiling, the system is open and the temperature of system rises to 100 °C. During steam cooking, the lid of container is tightly closed and cooking is completed under pressure and high temperature. Under these conditions, volatilization, hydrolysis, and thermal degradation may reduce pesticide residues. Our findings are consistent with previous studies (27, 28). Briefly, our results indicated that the removal of pesticide residues in this study was associated with both physicochemical properties of pesticides and cooking conditions. 

## Conclusion

 Pesticide residue in various commodities including rice is a major challenge in food safety. Rice is cooked in different methods all around the world and this study confirmed that Iranian home cooking processes reduced residue levels in contaminated rice. Washing and cooking removed different portions of pesticide residues in the range between 12.0-88.1% and 20.7-100%, respectively. The residue reductions were not correlated with chemical structure or water solubility of the pesticides but intensity of pesticide bindings to rice matrix, volatilization, hydrolysis, and thermal degradation determined the amounts of residues removed. In conclusion, proper home processing of rice before eating can result to reduction of intake of pesticide residues through rice consumption. 
